# Five years after the COVID-19 pandemic began: lessons on vaccination and obstetric care in Brazil

**DOI:** 10.61622/rbgo/2025rbgo94

**Published:** 2025-11-18

**Authors:** Renato Teixeira Souza, Maria Laura Costa, Jessica Lynn Schue, Emily Miller, Rupali Jayant Limaye, Jose Guilherme Cecatti

**Affiliations:** 1 Universidade de Campinas, Campinas Departmento de Tocoginecologia Campinas SP Brazil Departmento de Tocoginecologia, Universidade de Campinas, Campinas, SP, Brazil.; 2 Faculdade de Medicina de Jundiaí Jundiaí SP Brazil Faculdade de Medicina de Jundiaí, Jundiaí, SP, Brazil.; 3 Johns Hopkins University Bloomberg School of Public Health Department of International Health Baltimore USA Department of International Health, Bloomberg School of Public Health, Johns Hopkins University, Baltimore, USA.

**Keywords:** COVID-19, Vaccination, Pandemic, Obstetric care

## Introduction

The COVID-19 pandemic marked a defining moment in global health, laying bare the vulnerabilities of our healthcare systems and populations. For the obstetric population, the crisis posed unique and urgent challenges.

There was an immediate and marked rise in the maternal mortality ratio (MMR) and the perinatal mortality rate soon after the pandemic began, increasing by 33 % and 3.2 %, respectively. These indicators remained elevated throughout the pandemic period, with upward trends in caesarean delivery rates and preterm births. Access to antenatal, intrapartum, and postpartum services was compromised—especially at the pandemic's outset—due to resource limitations, movement restrictions, fear of contamination, and reorganisation of hospital workflows. This led to extra delays in diagnosing and treating obstetric complications, contributing to worse outcomes.^(1-3)^ Women from socially vulnerable groups (low education, non-white ethnicity, antenatal care via the public health system) faced higher risks of progressing to severe COVID-19 and severe acute respiratory syndrome (SARS), as well as greater exposure to risk factors such as obesity, hypertension, and diabetes.

Pregnant women were swiftly recognized as a high-risk group for severe outcomes from SARS-CoV-2 infection, including increased intensive care unit (ICU) admissions, need for mechanical ventilation, and higher maternal mortality. These realities transformed maternity wards into frontline environments of public health defence. Rapid reorganization of services was required, including bed reallocations, equipment acquisition, new protocols implementation, and increased workloads for health professionals. Many health professionals reported psychological distress, fear of infection, uncertainty, and lack of institutional support.^(2,4)^ Despite some advances—such as increased adoption of WHO-recommended practices (offering diet, non-pharmacological pain relief, early breastfeeding), medicalization and interventionist practices continued to predominate, although exacerbated by the pandemic context, especially at the beginning (pre-vaccine period).^(5)^

Social isolation, restrictions on companions, and disruption of support networks negatively affected the social lives and mental health of pregnant and postpartum women, with frequent reports of anxiety, fear of death, psychological suffering, and stigmatization.^(1,6)^ Early in the pandemic, COVID-19 testing was limited and prioritized for severe cases, hindering proper management and implementation of isolation and protective measures.^(3,4)^ More than half of the pregnant women evaluated in university hospitals in São Paulo state were found to have a higher likelihood of PTSD (Post-Traumatic Stress Disorder), especially those with anxiety symptoms, abrupt changes in routine, and contact with confirmed COVID-19 cases.^(7)^ Symptoms consistent with postpartum depression affected 38.8 % of the assessed women, with associated risk factors including anxiety, absence of a partner, and concerns about hospital bed availability. Suicidal ideation was reported by 14.3 % of the women and was strongly associated with anxiety and obtaining information via social media or friends.^(8)^ Social isolation, disruption of support networks, and fear of the disease were also highlighted as factors that worsened psychological distress, leading to frequent presentations of anxiety, depression, and discomfort with motherhood.^(1,6,9)^ The prevalence of intimate partner violence (IPV) was high among socioeconomically vulnerable women, particularly those living below the poverty line. In a study conducted in the Northeast Brazil, 22 % of women reported IPV, and exposure to violence was associated with more than double the odds of common mental disorders such as anxiety and depression. Job loss and food insecurity during the pandemic exacerbated both violence and negative mental health outcomes, imposing a dual burden on mothers.^(10,11)^

The role of the health providers in caring for the obstetric population in Brazil during the COVID-19 pandemic was multifaceted and essential for maintaining maternal care and addressing clinical, organizational, and ethical challenges. These professionals worked on the front lines, adapting clinical and organizational practices to ensure the safety of pregnant women, postpartum patients, and newborns, while also seeking to protect themselves and their teams and their own families, as highlighted in international guidelines and national experiences.^(2,4,12,13)^

In the Brazilian context, contingency plans were rapidly implemented in maternity wards, including the reorganization of patient flows, bed reallocations, acquisition of ventilators, and, to a lesser extent, the installation of negative-pressure rooms and temporary ICUs. COVID-19 testing was limited and prioritized for moderate to severe cases, reflecting resource and infrastructure constraints. Additionally, extra staff were hired and working hours were increased, with vacation suspensions to guarantee continuity of care.^(2,4)^

The pandemic also impacted care practices. Organizational changes, such as restrictions on companions and visits, were implemented to reduce transmission risk, but, in some cases, ran counter to the principles of humanized childbirth—particularly in regions with greater resource limitations.^(4)^

Healthcare professionals experienced marked psychological distress, reporting overload, fear of contagion, concern for their families, and insecurity in the face of disease uncertainties, all worsened by limited institutional support.^(2,4)^ Strict use of personal protective equipment (PPE), training in donning and doffing, screening of symptomatic pregnant women, and isolation of suspected cases were essential strategies to mitigate nosocomial transmission.^(12,13)^

Of note, the impact was not limited to the obstetric population. In the gynecological sphere, there was overall a significant reduction in the number of consultations—especially for screening, contraception, and reproductive planning—accompanied by a proportional increase in visits for benign conditions such as abnormal uterine bleeding and endometriosis. This shift reflects the prioritization of urgent cases and the limited access to preventive care during the pandemic.^(14)^

This health emergency highlighted the pivotal responsibility of obstetricians in reducing maternal morbidity and mortality, emphasizing the indispensable role of science-driven action and evidence-based practice. They assumed a critical role in this context—beyond the provision of clinical care; they played a central role in reorganizing services, implementing evidence-based protocols, advocating for the continuation of essential reproductive and maternal health care, and supporting their teams in the face of unprecedented challenges posed by the COVID-19 pandemic.^(2,5,12-14)^

### Scientific Engagement in Maternal and Perinatal Health During the Pandemic

Throughout the pandemic, the obstetric community's reliance on research and data was instrumental in informing responses. Clinical guidelines were in constant evolution, but the mobilization of collaborative research networks provided clarity in a rapidly shifting landscape. The FEBRASGO (Brazil´s National Ob&Gyn Federation) and the Ministry of Health (MoH; through the Primary Health Care Secretariat and the Department of Programmatic and Strategic Actions) launched a special working groups early in the pandemic, in 2020, to develop and promote evidenced-based guidelines for the assistance of pregnant and post-partum women with COVID-19 infection.^(15,16)^ Their endorsement of scientific recommendations and their personalized approach to patients’ fears were vital in promoting adherence to evolving public health guidance. The working groups’ task, while scientifically rigorous, did not escape the effects of the pandemic's politicization and competed with politics positions and endorsements of non-evidenced-based strategies and recommendations. Healthcare providers, especially obstetricians, became anchors of trust amid widespread misinformation. This underscores a broader lesson: the future of resilient maternal care is inextricably tied to the integration of research into daily practice.

### Spotlight on National Research Initiatives

The *REBRACO* initiative (Rede Brasileira de Estudos em COVID-19 em Obstetrícia; Brazilian Network of Obstetrical Studies on COVID-19 in Obstetrics) exemplifies the capacity of Brazilian medical professionals and scientists to lead in times of crisis. This collaborative multicenter prospective study in 16 Brazilian maternity hospitals comprehensively assessed SARS-CoV-2 infection during pregnancy and postpartum across five domains— encompassing qualitative studies, cross-sectional surveys, prospective cohorts, biobanking, and ecological studies, using standardized electronic data capture, robust statistical methods, and thematic qualitative analysis to inform pandemic response strategies.^(17)^ The main contributions were:

1. The REBRACO mixed-methods investigation described how 16 maternity hospitals rapidly activated contingency plans—reallocating beds, acquiring ventilators, hiring extra staff, and modifying leave policies—yet faced testing limitations (only one center universally screened) and challenges in infrastructure and human resources; qualitative interviews with managers underscored pervasive fear, evidence uncertainty, resource scarcity, and a critical need for mental health support among frontline teams.^(2)^
2. A year-long prospective cohort of 729 symptomatic pregnant and postpartum women across 15 Brazilian centers revealed that only half of suspected cases were tested early on, coinciding with peaks in ICU admissions and maternal deaths; confirmed COVID-19 was more common among socially vulnerable women and those with comorbidities (e.g., obesity, hypertension, diabetes), who exhibited significantly elevated risks for severe acute respiratory syndrome.^(3)^
3. In a nested case-control analysis of 203 pregnant women with COVID-19, preeclampsia prevalence (10.3 %) did not differ significantly from non-infected cohorts, but chronic hypertension and obesity markedly increased PE risk; PE cases underwent more caesarean deliveries (RR 5.54) and saw higher neonatal ICU admissions (PR 2.46), largely driven by prematurity.^(18)^
4. In a two-center prospective cohort of 170 vaccinated pregnant women with hypertensive disorders—31 of whom contracted COVID-19 during pregnancy and 139 who did not—there were no significant differences in sociodemographic profiles, the trajectory of the sFlt-1/PlGF ratio (using ≥ 38 to predict preeclampsia), or maternal and perinatal outcomes (notably high caesarean and preterm birth rates but zero maternal or neonatal deaths). These findings indicate that SARS-CoV-2 vaccination in this high-risk group yields comparable biomarker levels and clinical outcomes whether or not women experience COVID-19, underscoring the vaccine's protective effect against COVID-associated preeclampsia-like syndromes.^(19)^
5. Among 202 SARS-CoV-2–positive pregnant women stratified by pre-pregnancy BMI, obesity and overweight status were independently linked to worse clinical trajectories—including higher rates of sepsis, ARDS, and mechanical ventilation—and, after adjustment, obesity emerged as a strong predictor (aOR 3.73) of adverse perinatal outcomes.^(20)^
6. Through in-depth telephone interviews with 27 pregnant/postpartum women and six family members, this qualitative study uncovered that, before vaccine availability, COVID-19 suspicion or diagnosis triggered profound fear of death, hospitalization, quarantine, and financial strain, compounded by stigma from healthcare workers and social circles, resulting in significant physical, psychological, and socioeconomic impacts.^(6)^
7. In a nested case-control analysis of 481 unvaccinated pregnant women with respiratory symptoms, 43.7 % experienced adverse perinatal outcomes, which were independently associated with severe maternal illness (RR 3.30), residence in Brazil's North/Northeast (the most deprived regions in Brazil) (RR 3.09), and preeclampsia (RR 2.77), highlighting the imperative for timely, adequate care for symptomatic women regardless of COVID-19 confirmation.^(21)^
8. 8- A secondary analysis of the REBRACO cohort showed that COVID-19–positive pregnant women had a 22.4 % incidence of small-for-gestational-age newborns versus 14.8 % in test-negative peers, and that SGA infants born to infected mothers faced 1.6-fold higher odds of prematurity, stillbirth, neonatal death, or ICU admission—underscoring COVID-19's role in elevating neonatal morbidity and mortality.^(22)^
9. In a prospective cohort ancillary analysis of 91 placentas from SARS-CoV-2–positive pregnant women (versus 42 from test-negative controls), maternal vascular malperfusion lesions were significantly more frequent in the COVID-19 group, particularly following first- and second-trimester infections, while fetal vascular malperfusion and low-grade chronic villitis were not elevated. Although severe placental damage (COVID-19 placentitis) was rare, decidual arteriopathy correlated with early-pregnancy infections and chorangiosis with asymptomatic cases, suggesting that infection timing and clinical severity influence placental morphology changes.^(23)^
10. Comparing placentas from 14 SARS-CoV-2–infected women to 13 uninfected controls, researchers found no global increase in classical senescence markers but did observe elevated secretion of senescence-associated secretory phenotype (SASP) factors. Confocal imaging localized oxidative stress and DNA damage foci to areas expressing viral spike protein, indicating that COVID-19 can induce focal placental injury without widespread cellular senescence, underscoring the need to explore its clinical implications for maternal and neonatal outcomes.^(24)^
11. In a subanalysis of the multicenter REBRACO cohort including 285 pregnant women with confirmed COVID-19 (120 Black, 165 non-Black), Black women—who also had significantly lower education levels—showed similar delays in care but faced markedly worse maternal outcomes: they had higher odds of severe acute respiratory syndrome (OR 2.22, 95% CI 1.17–4.21), ICU admission (OR 2.00, 95% CI 1.07–3.74), and desaturation at admission (OR 3.72, 95% CI 1.41–9.84), and experienced greater maternal mortality (7.8 % vs 2.6 %, p = 0.048), while perinatal outcomes were comparable between groups.^(25)^

Considering that many specialists comprise the national working groups from FEBRASGO and the MoH, these findings have contributed to shaping national recommendations, especially those related to timely testing and vaccination priority (risky group), risk stratification by comorbidities and social vulnerability, equity in access to care, and improving clinical vigilance across diverse maternity settings during the pandemic.

Other academic initiatives have also played a crucial role in advancing maternal and perinatal health in Brazil. One notable example is the Brazilian Obstetric Observatory (Observatório Obstétrico Brasileiro – OOBr),^(26)^ originally conceptualized in 2018 by researchers and faculty from the University of São Paulo (USP) and the Federal University of Espírito Santo (UFES). The project aims to monitor and analyze public data based on the SIVEP-Gripe surveillance system. The SIVEP-Gripe is a national surveillance system managed by the Brazilian Ministry of Health, primarily used to monitor and record cases of severe acute respiratory infections, including those caused by influenza viruses and other respiratory pathogens like SARS-CoV-2 (COVID-19). With the involvement of various institutions, including students, professors, and researchers, the OOBr initiative offers interactive epidemiological dashboards that allow users to explore data at both state and municipal levels.

OOBr consolidates and publishes indicators from multiple data sources, such as the Mortality Information System (SIM), the Influenza Epidemiological Surveillance System (SIVEP-Gripe), the Live Birth Information System (SINASC), and vaccination records from the National COVID-19 Immunization Campaign. In response to the COVID-19 crisis, the OOBr COVID-19 panel was launched in April 2021 to provide timely information on maternal and perinatal health outcomes related to the pandemic. In 2022, the panel revealed that maternal mortality in Brazil was approximately 35% higher than the official figures reported by the Ministry of Health. To date, the group has contributed to the scientific literature with over ten significant publications addressing the pandemic's impact on obstetric populations.

Another dedicated group of Brazilian scientists leveraged SIVEP-Gripe, the national compulsory reporting system for severe acute respiratory syndromes, to build one of the most detailed epidemiological profiles of COVID-19 in the obstetric population worldwide.^(27-32)^ Through a series of complementary analyses using tens of thousands of cases from across the country, they consistently demonstrated that pregnant and especially postpartum women faced a disproportionately high risk of severe outcomes, including ICU admission, invasive ventilation, and death. Across studies, the postpartum period emerged as a critical window of vulnerability, with lethality rates often more than double those during pregnancy. Clinical risk factors such as obesity, diabetes, cardiovascular disease, and age over 35 were repeatedly identified, while social determinants—Black ethnicity, living in peri-urban areas, long distances from referral hospitals, and lack of access to primary health care—were shown to exacerbate risks. Strikingly, a substantial proportion of women who died never received ICU care or mechanical ventilation, underscoring persistent barriers to timely, high-quality treatment. Together, these investigations not only quantified the heavy toll of COVID-19 on Brazilian pregnant and postpartum women but also exposed deep inequities in healthcare access, offering an urgent call to action for targeted protective measures and improved maternal health infrastructure.

### Highlighting International Research: The WHO Multicountry Cohort

A milestone achievement in global research was the WHO-coordinated multicountry pregnancy cohort study, implemented across 10 countries, including Brazil. This unprecedented effort monitored over 16,000 pregnant and postpartum women, providing robust data on the impacts of COVID-19 infection and vaccination on maternal and neonatal outcomes (protocol available at Generic protocol: a prospective cohort study investigating maternal, pregnancy and neonatal outcomes for women and neonates infected with SARS-CoV-2, 1 November 2022 (who.int)). The study illuminated the operational challenges of conducting research during a pandemic and underscored the need for preparedness protocols and streamlined ethics review processes. It serves as a blueprint for research activation during future health emergencies.

### Vaccination: A Pillar of Public Health Policy

Brazil's history of successful public health programs—such as the National Immunization Program—was pivotal during the pandemic.^(33)^ The beginning of vaccination for pregnant women was associated with a reduction in the maternal mortality ratio, but other indicators, including caesarean rates, preterm birth and perinatal mortality ratio, remained unaltered compared to the pre-pandemic period.^(34)^ However, political polarization, misinformation, and systemic inequities posed obstacles to the COVID-19 vaccine rollout, especially for pregnant women. Misinformation about COVID-19 vaccination among pregnant and postpartum women in Brazil proliferated in an infodemic and politically polarized environment, driven by social media–spread rumors and fake news—including recycled Zika vaccine–microcephaly fears—that stoked safety concerns in expectant mothers;^(1,35)^ many struggled to discern reliable from false information amid politicization and distrust of authorities and traditional media.^(36,37)^ Ambiguous or anti-vaccine messaging from certain political leaders and religious figures further fuelled hesitancy.^(38)^ Institutional health communication often remained reactive and fragmented—focusing more on criticizing antivaccine movements than on proactively providing clear, accessible vaccine safety information for pregnant women.^(39)^ Pandemic-related disruptions to antenatal care increased reliance on digital sources of varying credibility and revealed gaps in vaccination counselling during clinical visits, especially in the private sector.^(1,35)^ This confluence of factors highlights the urgent need for coordinated health communication strategies that prioritize trusted sources and proactive engagement with the obstetric population.^(37,39)^

### Demand Generation study (Brazil)

Despite being prioritized from May 2021, COVID-19 vaccine hesitancy persisted. The World Health Organization (WHO) classified vaccine hesitancy and refusal as one of the ten threats to global health, given the growing vaccine hesitancy movement worldwide.^(40)^ Vaccine demand generation research can provide critical information and knowledge gaps for vaccine decision-making, including aspects of vaccine hesitancy. Vaccine decision-making is further complicated amongst certain populations and specific vaccines, including pregnant women and vaccines used in pregnancy.^(41)^ Specific information about this population can inform and support better communication between providers and this vulnerable population.^(42)^

A mixed-method multi-country investigation into COVID-19 vaccine attitudes and uptake among pregnant and postpartum women was implemented by the Johns Hopkins University research group, the World Health Organization experts, and international researchers from four countries.^(43)^ Drawing on both quantitative surveys and in-depth qualitative interviews conducted in Brazil, Ghana, Kenya, and Pakistan, the initiative revealed a complex interplay of individual beliefs, social influences, and policy contexts shaping vaccination decisions.^(44,45)^ In Brazil—where uptake was the highest among participating countries—women frequently cited self-protection and protecting their baby as primary motivators, and greater perceived risk of COVID-19, trust in vaccine safety and effectiveness, and the perception that peers were getting vaccinated were all associated with higher acceptance. Across settings, common barriers included safety concerns for both mother and child, low disease risk perception, and, in some cases, social or policy pressures. Healthcare providers emerged as trusted sources capable of countering hesitancy, while interpersonal influences, especially from partners, and broader community norms also played a role. Together, these studies underscored that increasing vaccine uptake in pregnancy requires not only clear, evidence-based public health messaging but also supportive environments that address safety concerns and leverage trusted social networks.

Surveying over 400 women in two Brazilian cities, this international initiative found that only a minority received a COVID-19 vaccine during pregnancy (manuscript submitted); More than two-thirds of the study population (71.3%) missed vaccination during pregnancy, although additional dose(s) would be recommended. Hesitancy was strongly linked to doubts about vaccine safety, effectiveness, and the severity of COVID-19, as well as preferences for "natural" immunity. Confidence in vaccination was significantly higher among women who relied on official information sources—such as healthcare professionals and public health agencies—than among those using non-official channels. These women reported greater trust in scientific information, more encouragement from family, and a higher likelihood of receiving multiple doses, including during pregnancy. Across both studies, the central message was clear: improving maternal COVID-19 vaccine uptake in Brazil requires not only addressing safety perceptions and misinformation, but also strengthening the role of trusted health professionals as key messengers.

Our results demonstrated that women who relied on official sources—such as healthcare providers or the government—were more likely to be vaccinated during pregnancy and have greater confidence in vaccine safety. These insights reinforce the central role of the obstetrician in vaccine counselling.

COVID-19 vaccination in the obstetric population in Brazil is of fundamental importance, both for maternal protection and for reducing adverse perinatal outcomes. Several national studies have shown that vaccination significantly reduces the risk of severe disease, ICU admission, need for ventilatory support, maternal sepsis, and maternal mortality, even in the face of circulating variants of concern.^(34,46-48)^

The effectiveness of vaccination—especially with a complete scheme—has been demonstrated in preventing severe forms of the disease in pregnant women, with data showing that two doses of CoronaVac, for example, confer robust protection against symptomatic COVID-19 and, particularly, against severe cases.^(49)^ In addition, maternal vaccination is associated with reduced neonatal admissions and respiratory complications in newborns.^(47)^ At the population level, time-series analyses indicate that, after the implementation of vaccination in pregnant women, there was an immediate and sustained decrease in Brazil's maternal mortality ratio, reversing part of the increase observed at the pandemic's outset.^(34)^ National ecological studies have also confirmed the significant reduction in COVID-19–related maternal mortality following the start of vaccination, even in regions with high social vulnerability.^(48)^

Regarding safety, Brazilian data indicate that adverse events following vaccination in pregnant and postpartum women are, for the most part, mild and self-limiting, without an increased risk of adverse obstetric outcomes—such as preterm birth, low birth weight, or neonatal death—regardless of vaccine type (inactivated or mRNA) or trimester of administration.^(50,51)^ Therefore, COVID-19 vaccination is safe, effective, and directly impacts the reduction of maternal and neonatal morbidity and mortality in Brazil, representing a public health priority strategy for the obstetric population.^(46,49-51)^ Currently, the Brazilian Ministry of Health recommends a booster dose of the COVID-19 vaccine for pregnant women whose last dose was administered more than six months before pregnancy.^(52)^

A recent guideline launched in August 2025 by The Brazilian Society of Paediatrics, the Brazilian Society of Immunizations, and the Brazilian Federation of Gynaecology and Obstetrics endorsed the recommendation that pregnant and postpartum women be considered a priority group for COVID-19 vaccination, receiving one dose during each pregnancy.^(53)^ Vaccination is advised for all pregnant women, with those who have had COVID-19 waiting at least four weeks after symptom onset before receiving the vaccine. Any available mRNA-based COVID-19 vaccine may be administered during pregnancy, and for those not vaccinated while pregnant, immunization is recommended in the postpartum period and/or during lactation.

### Lessons Learned: Building a Resilient Future

The COVID-19 pandemic challenged and reshaped our health system. It also taught us invaluable lessons:

- Science and agility must coexist: Evidence-based practice needs to be accompanied by flexible, rapid responses during crises. The involvement of the academic community and medical societies, such as FEBRASGO, is fundamental to building robust and standardized recommendations.
- Trust is foundational: The obstetrician's voice is often the most trusted in a pregnant woman's life. Strengthening this relationship can combat misinformation.
- Preparedness is a professional obligation: Research and care readiness, ethical agility, and collaborative infrastructure must be in place before the next crisis. Obstetricians and gynaecologists must play a key role in this endeavour: building and fighting for women's equitable health care.

As we move forward, let us integrate these learnings into daily obstetric care, ensuring that no woman, no matter her background or circumstance, faces the journey of motherhood alone or unprotected.
